# Venetoclax combined chemotherapy versus chemotherapy alone for acute myeloid leukemia: a systematic review and meta-analysis

**DOI:** 10.3389/fonc.2024.1361988

**Published:** 2024-03-26

**Authors:** Jingkui Zhu, Jixin Fan, Tiantian Xie, Haiqiu Zhao, Runqing Lu, Yinyin Zhang, Yingmei Li, Xinsheng Xie, Dingming Wan, Zhongxing Jiang, Fei He, Rong Guo

**Affiliations:** ^1^ Department of Hematology, The First Affiliated Hospital of Zhengzhou University, Zhengzhou, China; ^2^ Department of Cardiology, The First Affiliated Hospital of Zhengzhou University, Zhengzhou, China

**Keywords:** meta-analysis, acute myeloid leukemia, venetoclax, chemotherapy, efficacy, adverse events

## Abstract

**Objective:**

To compare the efficacy and safety of venetoclax (VEN) in combination with chemotherapy (chemo) versus chemo alone in the treatment of acute myeloid leukemia (AML).

**Method:**

To compare the efficacy and/or safety of VEN+chemo versus chemotherapy alone for AML, PubMed, Embase, Web of Science, and the Cochrane Library were used to searching up to June 2023. Comparisons included complete remission (CR), CR with incomplete hematologic recovery (CRi), morphologic leukemia-free state (MLFS), overall response rate (ORR), and adverse events (AEs).

**Result:**

A total of 9 articles were included, including 3124 patients. The baseline characteristics between two patient groups were similar. The combined analysis showed that compared with the group receiving chemo alone, the VEN+chemo group exhibited higher rates of CR, CRi, MLFS and ORR. Additionally, the VEN+chemo group had longer event-free survival (EFS) and overall survival (OS) durations. The incidence rates of AEs and serious AEs (SAEs) were similar between the two groups, but the early 30-day mortality rate was lower in the VEN+chemo group than in the chemo alone group.

**Conclusion:**

The VEN+chemo therapy demonstrates significant efficacy and safety profile in AML patients. However, more prospective studies are needed in the future to provide more accurate and robust evidence for treatment selection in patients.

**Systematic Review Registration:**

https://www.crd.york.ac.uk/prospero/display_record.php?ID=CRD42023439288, identifier CRD42023439288.

## Introduction

Acute myeloid leukemia (AML) manifests as a remarkably heterogeneous hematological malignancy, marked by impediments in myeloid differentiation and aberrant proliferation of immature myeloid progenitor cells (1). With a median age of 68 years at diagnosis, AML emerges as the most prevalent form of acute leukemia in adults, and its incidence rises with age (2, 3).The current standard intensive induction therapy for newly diagnosed acute myeloid leukemia (ND-AML) is a 7 + 3 regimen comprising cytarabine in combination with anthracyclines, followed by consolidation therapy upon achieving remission. Elderly patients and individuals with substantial capabilities comorbidities are generally deemed inappropriate candidates for intensive chemotherapy (chemo). This frequently leads to a reduced response rate when subjected to low-intensity chemo protocols, such as those involving hypomethylating agents (HMA) and low-dose cytarabine (4). Moreover, the absence of standardized treatment protocols leads to a long-term survival rate of less than 20% and a bleak prognosis for relapsed or refractory AML (R/R-AML) (5). The majority of AML patients have limited opportunities for effective treatment options. Consequently, there is a pressing need for research and the development of more potent treatment strategies to improve patient prognosis.

Venetoclax (VEN) is a selective small molecule inhibitor of B cell lymphoma 2 (BCL-2), effectively interrupting BCL-2’s inhibitory effects on pro-apoptotic proteins BAX and BIM. It demonstrates anti-tumor activity against a range of hematologic malignancies by increasing the permeability of the mitochondrial outer membrane, facilitating the release of cytochrome C, and thereby inducing apoptosis (6). This study suggests that, compared to chemo alone, VEN+chemo can improve the prognosis of AML patients (7).Nevertheless, research also indicates that patients undergoing VEN+chemo have lower rates of complete remission (CR) and shorter overall survival (OS) compared to those in the chemo-alone group (8). Concurrently, there is controversy surrounding the question of whether VEN+chemo leads to an increased occurrence of adverse events (AEs) and/or serious AEs (SAEs) in patients (9, 10). Presently, a deficiency exists in accessible meta-analyses for comparing outcomes between the two groups. Consequently, we conducted a thorough systematic literature review and meta-analysis to evaluate the effectiveness and safety of VEN+chemo in comparison to chemo alone in AML patients.

## Method

This study was conducted according to the Preferred Reporting Items for Systematic Evaluation and Meta-Analysis (PRISMA statement) and registered in the PROSPERO International Registry of Prospective Systematic Reviews (registration number: CRD42023439288).

### Literature search

Until June 2023, we conducted an extensive literature search utilizing multiple databases (PubMed, Embase, Web of Science, and the Cochrane Library) to compare the efficacy and safety of VEN+chemo to chemo alone for AML patients. The search terms used were “venetoclax,” “chemotherapy,” and “Acute Myeloid Leukemia”. The comprehensive search strategy is outlined in **Supplementary Data Sheet 1**. Additionally, we manually reviewed the reference lists of all eligible studies. Two investigators (HZ and RG) independently retrieved and evaluated the selected studies, resolving any discrepancies in the literature search through collaborative consensus.

### Inclusion and exclusion criteria

The inclusion criteria were as follows (1): the study design encompassed cohort or case-control, and randomized controlled trial (RCT) (2);adult patients with AML were involved in this study (3); study comparing the combination of VEN with chemo to chemo alone; and (4) the study reported outcome metrics, such as efficacy and AEs.

Exclusion criteria were as follows (1): reviews, meta-analyses, letters, editorial comments, case reports, conference abstracts, pediatric articles, unpublished articles, animal studies, non-English language articles (2); duplicate publications.

### Data extraction

The two investigators (JZ and JF) independently extracted the data, which included (1) basic information of the included studies, such as authors, year of publication, type of study, sample size, intervention, etc. (2); basic characteristics of the study subjects, such as median age, gender, etc.; and (3) outcome metrics, such as CR, CR with incomplete hematologic recovery (CRi), morphologic leukemia-free state (MLFS), and AEs. In case of disagreement, a third investigator (TX) was involved in the discussion to resolve it.

### Quality assessment

The quality of the included cohort studies was assessed according to the Newcastle-Ottawa Scale (NOS), a frequently employed tool for assessing the quality of observational studies. The NOS examines the potential bias stemming from the selection of study participants, misclassification, and confounding in association measurements. Studies scoring 7-9 points are generally regarded as high quality, whereas those scoring 3 or lower are deemed low quality. Furthermore, the quality evaluation of randomized controlled trials (RCTs) was carried out through the Cochrane Risk of Bias Assessment tool (11). Which is widely used for evaluating the quality of RCTs and primarily examines bias risks in several domains including random sequence generation, allocation concealment, blinding, completeness of outcome reporting, management of incomplete outcome data, and other possible biases. The utilization of this tool enables reviewers to develop a thorough comprehension of bias in RCT studies, facilitating an effective assessment of research quality and result reliability. The quality and level of evidence of the eligible studies were assessed independently by two researchers (JZ and JF), and any disagreements were resolved through discussion (TX).

### Statistical analysis

Statistical analyses were performed using RevMan 5.3 software. Heterogeneity of the studies was assessed by χ2 and I^2 (heterogeneity was considered significant when χ2 *P* < 0.05 or I^2>50%), and if the heterogeneity was significant, a random-effects model was used, otherwise a fixed-effects model was used. Odds ratio (OR) were used to compare categorical variables and hazard ratio (HR) were used to compare survival variables and 95% confidence intervals (CI) were reported. Publication bias was assessed by funnel plot and Egger regression test. In addition, we performed subgroup analyses and one-way sensitivity analyses for outcomes with significant heterogeneity. For the other tests, *P* < 0.05 was considered statistically significant.

## Results

### Literature search and characteristics

**Figure 1:**
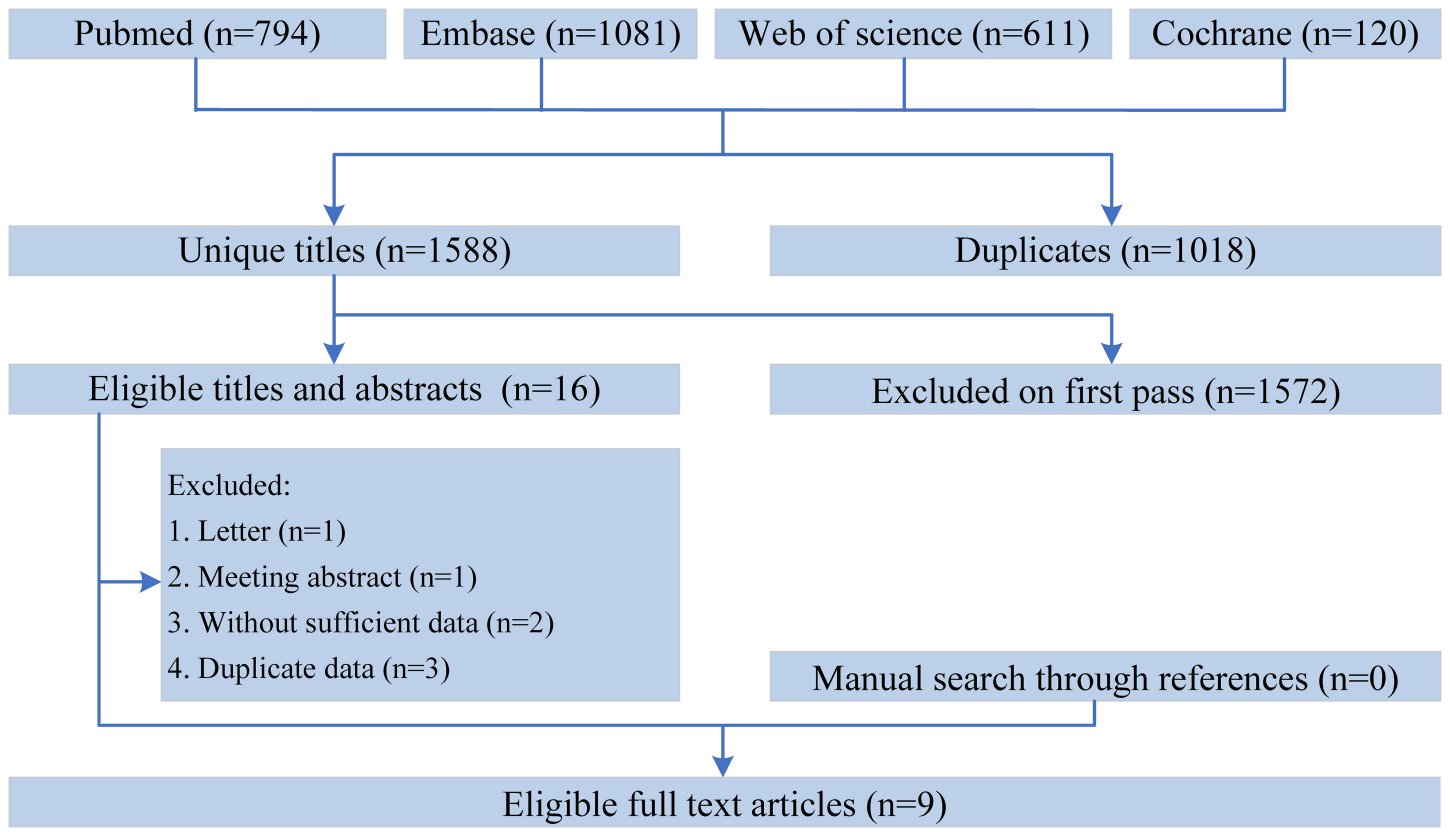
The flow chart of the selection process.

Initially, a preliminary search was performed on a total of 2,606 articles relevant to the study (794 articles from PubMed, 1,081 from Embase, 611 from Web of Science, and 120 from the Cochrane Library). Following the removal of duplicate papers, a comprehensive screening of titles and abstracts for1,588 papers was conducted. Eventually, a total of 9 articles were selected, consisting of data from 3,124 patients (5, 8–10, 12–16). Of these, seven were cohort studies and two were RCTs. The flow chart of the selection process was shown in **Figure 1**. **Table 1** exhibits the baseline characteristics of included studies. The cytogenetic/molecular/ELN risk information listed in **Supplementary Table S1**.

**Table 1:** Baseline characteristics of include studies and methodological assessment.

Authors	Study period	Country	Study design	Types of AML	Patients (n)	Median age(years)	Male(n)	Median follow-up	Combination chemotherapy regimen	Chemotherapy alone	Quality score
					Venetoclax+chemo/Chemo	Venetoclax+chemo/Chemo	Venetoclax+chemo/Chemo				
Cherry (8)	2007-2020	USA	Cohort	Newly diagnosed AML	143/149	68.35/52.47	72/78	4.65years	The simultaneous combination of ven and aza at any dose or schedule, and 149 received IC, defined as a multiday cytarabine-containing regimen at $100 mg/m^2^ per day	Intensive chemotherapy	7
DiNardo (10)	2017-2019	USA	RCT	Previously untreated patients with confirmed AML who were ineligible for standard induction therapy	286/145	75.67/75.91	172/87	20.5 months	Venetoclax (Venetoclax was administered orally, once daily, with food. For mitigation of the tumor lysis syndrome during cycle 1, the dose of venetoclax was 100 mg on day 1 and 200 mg on day 2; on day 3, the target dose of 400 mg was reached and continued until day 28) + azacitidine (5 mg per square meter of body-surface area, subcutaneously or intravenously, on days 1 through 7 every 28-day cycle)	Placebo + azacitidine (5 mg per square meter of body-surface area, subcutaneously or intravenously, on days 1 through 7 every 28-day cycle)	NA
Gershon (12)	2014-2021	USA	Cohort	Newly diagnosed AML	619/480	74.80/75.80	383/295	6.3 months	Venetoclax + HMAs (Aza ± hydroxyurea, or Dec ± hydroxyurea)	HMA monotherapy (Aza ± hydroxyurea, or Dec ± hydroxyurea)	6
Kwag (13)	2013-2021	Korea	Cohort	Newly diagnosed AML	74/74	71.35/72.71	32/37	8.5 months	The DEC+VEN group received the same daily dose of DEC for five days combined with 28 days of VEN (400 mg daily) per cycle.	DEC monotherapy consisted of administering 20 mg/m2 intravenous DEC daily for five days.	8
Lachowiez (14)	2010-2021	USA	Cohort	Newly diagnosed AML	85/194	46.06/49.60	44/87	30 months	Venetoclax + IC (FLAG-IDA or CLIA)	IC (FIA, CLIA, or CIA)	8
Maiti (5)	2005-2020	USA	Cohort	Relapsed or refractory AML	65/130	62.14/57.20	39/72	49.3 months	Decitabine 20 mg/m2 daily for 10 days every 4 to 6 weeks with venetoclax 400 mg daily or equivalent (with concomitant azole antifungals) for induction.	IC (salvage therapy with idarubicin with cytarabine (IA), with or without cladribine (CLIA), clofarabine (CIA), or fludarabine (FIA or FLAG- IDA)– based regimens)	9
Maiti (15)	2000-2019	USA	Cohort	Newly diagnosed AML	85/85	73.06/71.94	45/48	81.2 months	The regimen comprised of daily venetoclax with decitabine 20 mg/m2 IV for 10 days for “induction”, followed by decitabine for 5-days as consolidation. Venetoclax dose was 400 mg daily or equivalent with concomitant azoles.	Intensive chemotherapy containing at least moderate dose of cytarabine 1 g/m2/d	8
Park (16)	2018-2021	Korea	Cohort	Relapsed or refractory AML	54/89	49.41/48.58	24/52	22.5 months	VEN with HMA (VEN/HMA) or with LDAC (VEN/LDAC) was administered in 28-day cycles.	IC (MEC, FLANG, or FLAG-IDA)	8
Wei (9)	2017-2019	Multicenter	RCT	AML who had not received prior AML treatment and were ineligible for intensive chemotherapy	142/68	74.99/74.34	78/39	17.5 months	Venetoclax (100 mg venetoclax orally on day 1, 200 mg on day 2, 400 mg on day 3, and 600 mg daily on days 4–28 of cycle 1 and daily in all subsequent 28-day cycles) + LDAC (20 mg/m2 subcutaneously on days 1–10 of each 28-day cycle)	Placebo + LDAC (20 mg/m2 subcutaneously on days 1–10 of each 28-day cycle)	NA

AML, acute myeloid leukemia.

### Quality assessment and risk of bias

Of the 7 cohort studies, 6 were high quality studies with a score of 7-9 (**Table 1**). Details of the quality ratings of all eligible cohort studies are provided in **Supplementary Table S2**. The quality ratings of the 2 RCT studies were shown in **Supplementary Figure 1**.

### Treatment response

**Figure 2:**
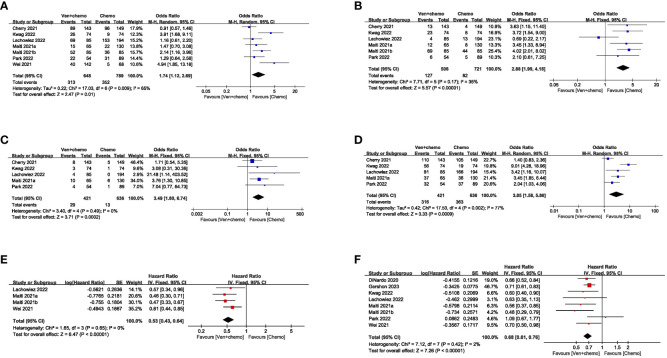
Assessment of heterogeneity in outcome measures.

**Figure 3:**
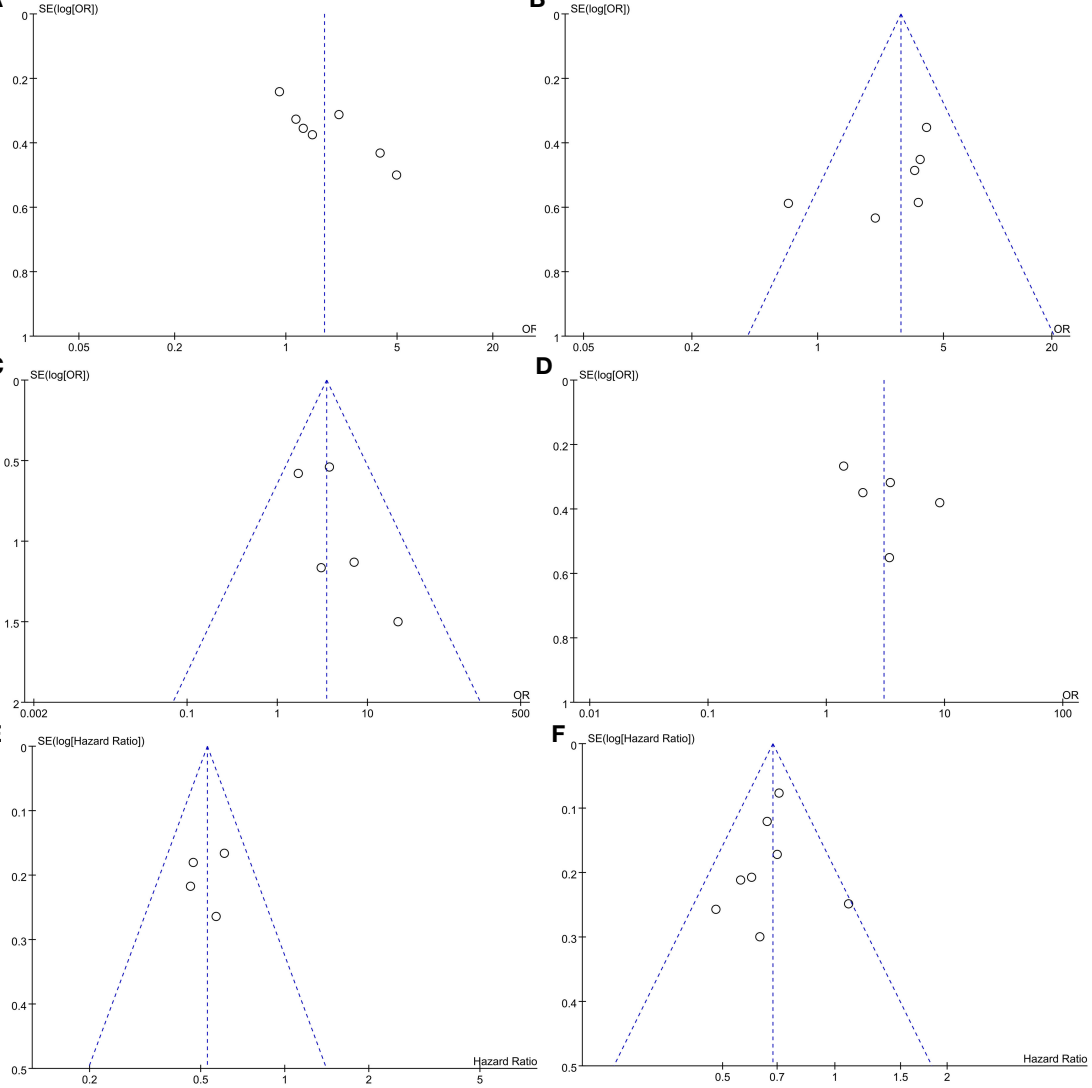
Funnel plot of outcome measures in meta-analysis.

1. CR: Seven studies reported the CR of patients, and the results revealed that the CR was higher in the VEN+chemo group compared to the chemo alone group (48.3% vs 44.6%). The combined effect was statistically significant (OR=1.74, 95%CI: 1.12-2.69), and significant heterogeneity (I^2 = 65%, *P*=0.009) depicted in **Figure 2A**. Without obvious publication bias exhibited in Funnel plot (**Figure 3A**), but the Egger’s test with (*P*=0.017);
2. CRi: The meta-analysis results of CRi in patients from six studies indicated that CRi was higher in the VEN+chemo group compared to the chemo alone group (25.1% vs 11.4%). The combined effect was statistically significant (OR=2.88, 95%CI: 1.99-4.18). The study results showed heterogeneity (I^2 = 35%, *P*=0.17), as shown in **Figure 2B**. Publication bias was observed in the funnel plot (**Figure 3B**), but not in the Egger’s test (*P*=0.193);
3. MLFS: Five studies used MLFS to assess treatment efficacy, and there was no heterogeneity among the study results (I^2 = 0%, *P*=0.49). The study results indicated that the MLFS was higher in the VEN+chemo group compared to the chemo alone group (6.9% vs 2.0%, OR=3.49, 95%CI: 1.80-6.74) (**Figure 2C**). The funnel plot (**Figure 3C**) and the Egger’s test did not reveal any significant publication bias (*P*=0.203);
4. Overall response rates (ORR): The five included studies used ORR as the measure of therapeutic effect. The results showed that the ORR was higher in the VEN+chemo group compared to the chemo alone group (75.1% vs 57.1%), and the combined effect was statistically significant (OR=3.05, 95%CI: 1.58-5.86). There was significant heterogeneity in the study results (I^2 = 77%, *P*=0.002) (**Figure 2D**), the funnel plot (**Figure 3D**), and Egger’s test(*P*=0.355) had no obvious publication bias.

### Event-free survival and OS

(1) EFS: Seven studies reported the EFS of the patients. The results showed that the VEN+chemo group had a longer EFS compared to the chemo alone group, and the combined effect was statistically significant (HR=0.53, 95%CI: 0.43-0.64). There was no significant heterogeneity in the study results (I^2 = 0%, *P*=0.65), as depicted in **Figure 2E**. The funnel plot (**Figure 3E**), and Egger’s test (*P*=0.781) found no obvious publication bias;
(2) OS: Eight studies used OS as the evaluation measure. The results showed that the VEN+chemo group had a longer OS compared to the chemo alone group, and the combined effect was statistically significant (HR=0.68, 95%CI: 0.61-0.76). There was no significant heterogeneity in the study results (I^2 = 2%, *P*=0.42), as depicted in **Figure 2F**. The funnel plot (**Figure 3F**), and Egger’s test (*P*=0.551) exhibited no significant publication bias.

### Safety

We found that almost all patients experienced at least one AE (99%). The most prevalent AEs observed in both study groups included neutropenia, thrombocytopenia, nausea, and infection. Although the SAEs incidence in VEN+chemo group was higher than chemo alone group, but the difference was not statistically significant(*P*>0.05) in AEs and SAEs. Early 30-day mortality, of VEN+chemo group was superior to the chemo alone group (OR=0.23, 95%CI=0.12-0.48, *P*<0.0001).

### Subgroup analysis and sensitivity analysis

We performed subgroup analyses of efficacy measures (CR, CRi, MLFS, overall response, OS, EFS) according to different study design, combining scheme, region, and types of AML. The results indicate that the VEN+AZA, VEN+IC, Asia, America, and R/R-AML subgroups were unsatisfactory in some of the efficacy indices, while the other subgroups showed no significant changes (detailed analysis in **Table 2**). Specifically, the VEN+AZA group exhibited inconsistency with the overall results in CR, MLFS, and overall response; the VEN+IC group showed inconsistency in CR, CRi, and OS; the Asia and America groups were inconsistent with the overall results in CR; and the R/R-AML group showed inconsistency in CR and OS.

**Table 2:** Subgroup analysis.

Subgroup	CR				CRi				MLFS				Overall response				OS				EFS			
	Study	OR [95%CI]	*P* value	I^2	Study	OR [95%CI]	*P* value	I^2	Study	OR [95%CI]	*P* value	I^2	Study	OR [95%CI]	*P* value	I^2	Study	HR [95%CI]	*P* value	I^2	Study	HR [95%CI]	*P* value	I^2
**Total**	7	1.74 [1.12-2.69]	0.01	65%	6	2.88 [1.99-4.18]	<0.00001	35%	5	3.49 [1.80-6.74]	0.0002	0%	5	3.05 [1.58-5.86]	0.0009	77%	8	0.68 [0.61-0.76]	<0.00001	2%	4	0.53 [0.43-0.64]	<0.00001	0%
Study design																								
RCT	1	4.94 [1.85-13.18]	0.001	NA													2	0.67 [0.55-0.82]	<0.00001	0%	1	0.61 [0.44-0.85]	0.003	NA
Cohort	6	1.51 [1.02-2.25]	0.04	55%													6	0.68 [0.60-0.77]	<0.00001	29%	3	0.49 [0.38-0.62]	<0.00001	0%
Combining scheme																								
Venetoclax+azacitidine	1	0.91 [0.57-1.46]	0.7	NA	1	3.63 [1.15-11.40]	0.03	NA	1	1.71 [0.54-5.35]	0.36	NA	1	1.40 [0.83-2.36]	0.21	NA	1	0.66 [0.52-0.84]	0.0006	NA				
Venetoclax+decitabine	3	2.23 [1.34-3.69]	0.002	32%	3	3.79 [2.36-6.08]	<0.00001	0%	2	3.61 [1.37-9.49]	0.009	0%	2	5.45 [2.13-13.94]	0.0004	73%	3	0.55 [0.43-0.71]	<0.00001	0%	2	0.47 [0.35-0.61]	<0.00001	0%
Venetoclax+IC	1	1.16 [0.61-2.20]	0.66	NA	1	0.69 [0.22-2.17]	0.52	NA	1	21.48 [1.14-403.52]	0.04	NA					1	0.63 [0.35-1.13]	0.12	NA	1	0.57 [0.34-0.96]	0.03	NA
Venetoclax+cytarabine	1	4.94 [1.85-13.18]	0.001	NA													1	0.70 [0.50-0.98]	0.04	NA	1	0.61 [0.44-0.85]	0.003	NA
Region																								
Asia	2	2.18 [0.73-6.49]	0.16	75%	2	3.11 [1.52-6.34]	0.002	0%	2	4.75 [0.98-23.14]	0.05	0%	2	4.26 [1.00-18.22]	0.05	88%	2	0.77 [0.56-1.05]	0.09	71%				
America	4	1.31 [0.88-1.93]	0.18	39%	4	2.80 [1.81-4.33]	<0.00001	58%	3	3.23 [1.56-6.70]	0.002	31%	3	2.39 [1.21-4.68]	0.01	64%	5	0.67 [0.59-0.75]	<0.00001	0%				
Types of AML																								
Newly diagnosed	5	1.97 [1.06-3.66]	0.03	76%	4	0.35 [0.13-0.96]	0.04	0%	3	2.91 [1.17-7.23]	0.02	24%	3	3.44 [0.99-11.94]	0.05	88%	6	0.67 [0.60-0.75]	<0.00001	0%	3	0.55 [0.44-0.68]	<0.00001	0%
Relapsed or refractory	2	1.37 [0.83-2.27]	0.22	0%	2	2.89 [1.88-4.43]	<0.00001	59%	2	4.32 [1.67-11.17]	0.003	0%	2	2.71 [1.63-4.52]	0.0001	18%	2	0.74 [0.54-1.02]	0.06	76%	1	0.46 [0.30-0.71]	0.0004	NA

AML, Acute myeloid leukemia; CR, complete remission; CRi, CR with incomplete hematologic recovery; MLFS, morphologic leukemia-free state; EFS, Event-Free Survival; OS, overall survival; OR, odds ratio; CI, confidence intervals; HR, hazard ratio; IC, intensive chemotherapy.

**Figure 4:**
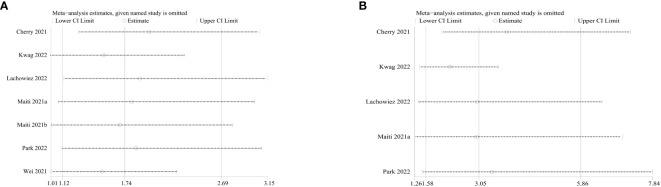
Sensitivity analysis of complete response (CR) and overall response rate (ORR).

In addition, we performed a one-way sensitivity analysis on CR and ORR, evaluating the influence of each study on the stability and heterogeneity of CR and ORR using the method of one-by-one exclusion. The results showed that the statistical differences in CR (**Figure 4A**) and ORR (**Figure 4B**) remained unchanged after excluding any individual literature, and significant heterogeneity still existed after excluding any individual literature.

## Discussion

VEN has significant anti-tumor activity against various hematologic malignancies, including AML. However, the efficacy and safety of VEN combined with chemo in AML patients are still controversial, more high-quality research still needed. To our knowledge, this is the first systematic review and meta-analysis comparing the efficacy of VEN combined with chemo versus chemo alone in AML patients. In this study, we conducted a meta-analysis of 3124 patients from 9 publications to resolve this clinical controversy. The results of this study showed that the VEN combined with chemotherapy group had significantly better treatment response rates and survival time than the chemo alone group. The CR, CRi, and ORR rates in the VEN combined with chemo group were 48.3%, 25.1%, and 75.1%, respectively. These findings are similar to the meta-analysis results of previous studies on VEN combined therapy for AML, further validating the effectiveness of combination therapy (17).

VEN-based combination regimens are currently approved for the treatment of ND-AML patients who are elderly or unsuitable for IC, but there is still a lack of studies in R/R-AML patients (18). The subgroup analysis results in this study showed that R/R-AML patients who received combination therapy had higher CR/CRi/ORR rates, which may be associated with the targeting and synergistic effects of VEN. AML cells, particularly leukemia stem cells, are dependent on BCL-2 for their survival. VEN’s inhibitory action has the capacity to stimulate intrinsic apoptosis pathways, resulting in the prompt induction of apoptosis in AML cells and the elimination of dormant leukemia stem cells. VEN possesses the ability to activate T cells directly, both *in vivo* and *in vitro*, thereby enhancing their cytotoxicity against AML. By inhibiting the formation of respiratory chain super complexes, VEN ultimately boosts the effector function of T cells by enhancing the generation of reactive oxygen species (19). VEN used alone may lead to drug resistance, highlighting the importance of combining it with other chemo drugs. The mechanism of VEN resistance is not yet clear, but it may be related to the RAS/MAPK/MCL-1 pathway, leading to the upregulation of anti-apoptotic BCL-2 family proteins (such as BCL-XL and MCL-1), which effectively enhance the survival of leukemia cells (20). The concurrent use of VEN with chemo can synergistically induce cell apoptosis, collaboratively trigger mitochondrial apoptosis in AML cells, lower MCL-1 levels, thereby overcoming resistance in AML, and heightening anti-tumor efficacy (19).

The study conducted by Lee et al. (19), as reported in BLOOD, elucidated that VEN exerts a direct enhancement on the anti-leukemic effector function of T cells. Conversely, azacitidine induces a type I interferon response by activating the STING-cGAS pathway, thereby eliciting a virus-like infection response in leukemia cells. The increased susceptibility of AML cells to T cell-mediated cytotoxicity is observed in this study. Notably, the treatment response rate in the group with ND-AML surpassed that in the group with R/R-AML. This could be attributed to T cell dysfunction following chemo in the group with R/R-AML and could additionally be associated with the heightened probability of R/R-AML patients harboring adverse prognostic chromosomal karyotypes and gene mutations (e.g., TP53, SF3B1, EZH2) that make them less responsive to VEN (21, 22). In this study, the efficacy of the VEN+AZA and VEN+IC subgroups was suboptimal. This may be attributed to the higher incidence of neutropenia in patients receiving combination therapy in the VEN+AZA group, leading to treatment interruption for hematologic recovery. Moreover, within the VEN+IC cohort, a greater percentage of patients receiving IC exhibited FLT3-ITD mutations. Consequently, patients receiving IC treatment also received FLT3 inhibitor therapy. However, heterogeneity exists in our study, and may related with the differences in types of AML and treatment protocols. Specifically, the response to VEN in ND-AML and R/R-AML patients varies. For instance, R/R-AML patients receiving combination therapy demonstrate improved treatment efficacy compared to those undergoing chemo alone. Additionally, the treatment response rate in the ND-AML group was higher than that in the R/R-AML group.

Different molecular features can significantly influence the efficacy of VEN. It has been reported that patients with mutations in NPM1, TET2, IDH1/2, ASXL1 and DDX41 have a higher response rate to VEN (23–30). In which, DDX41 is a DEAD-box type helicase that participates in various cellular processes including RNA metabolism and splicing (31). DDX41 mutations affect small nucleolar RNA maturation, impair ribosomal rRNA modification, hinder cellular protein synthesis, leading to cell cycle arrest and promoting apoptosis of mutated blood cells (32). In addition, splice factor (SF) mutations such as SRSF2, U2AF1, SF3B1, and ZRSR2 are commonly found in elderly AML patients and portend a poor prognosis (33). Lachowiez et al. (34) revealed that the outcome of patients with SF mutations treated with VEN+ hypomethylating agents was comparable to that of the wild-type patients. The improved prognosis of patients with DDX41 and SF mutations following VEN-based therapy treatment may be attributed to the potential influence of these mutations on the expression of BCL-2 family genes, thereby impacting the response to VEN-based therapy (35). It is worth noting that Stahl et al (23) found the mutation status of DNMT3A and the treatment history of HMA can predict the treatment response of patients with R/R-AML to VEN+HMA. For R/R-AML patients without DNMT3A mutations, regardless of previous HMA treatment, their survival rates after VEN+HMA therapy are similar. For R/R-AML patients with DNMT3A mutations who have not received prior HMA treatment, the response rate to VEN+HMA is higher, and their survival period is longer. Conversely, for R/R-AML patients with DNMT3A mutations who have a history of HMA treatment, the response rate to VEN+HMA is lower, and their survival period is shorter. Although it is not possible to conduct a quantitative analysis of median OS due to the different follow-up periods in each study, our research still indicates that VEN+chemo can prolong EFS and OS in AML patients. At the same time, the use of VEN-based combination therapy may improve the poor prognosis associated with certain genetic mutations. This discovery provides new possibilities for personalized treatment of AML patients.

The safety analysis results indicate that there were no significant differences in AEs and SAEs between the two groups of patients. Compared to chemo alone, VEN+chemo does not increase the incidence of AEs and/or SAEs in patients, and the early 30-day mortality rate was lower than the chemo alone group. Similar to the previous meta-analysis results, almost all patients experienced at least one AE during the study. The most common AEs in patients treated with the combination of VEN and chemotherapy were neutropenia, thrombocytopenia, nausea, and infection (17, 36). However, it is important to note that the safety assessment results are based on limited data. Therefore, in clinical practice, it is still necessary to consider individual differences in patients in order to better evaluate potential risks and benefits.

This study provides the first systematic comparison of the efficacy and safety between VEN-based combination therapy and chemo alone in AML patients. In order to ensure the reliability of the results, we employed a comprehensive search strategy, clearly defined selection criteria, conducted rigorous quality assessments, and reported according to the PRISMA statement. The study confirms the superiority of VEN-based combination therapy over chemo alone in AML patients. However, our study has the following limitations: First, this meta-analysis included seven cohort studies and two RCTs, lacking prospective studies, which may impact the reliability of the results. Therefore, more RCTs and prospective clinical studies are needed to confirm our findings. Second, the results in this meta-analysis exhibit high heterogeneity. Subgroup analysis and sensitivity analysis were performed to evaluate the sources of heterogeneity, but it is difficult to determine all the factors contributing to heterogeneity. Considering the potential confounders, the results of this meta-analysis should be interpreted with caution. Third, due to small sample sizes in some subgroup analyses, it was challenging to quantitatively synthesize the data, and larger sample size needed for further analysis. Fourth, influenced by the limitations of the original study, we were unable to assess safety outcomes such as cycle length and hospitalization rates in patients. Consequently, it is essential to conduct further research to thoroughly investigate these aspects in the future. Ultimately, the existing literature remains limited despite conducting comprehensive searches across multiple databases. It is important to acknowledge the potential presence of publication bias, as this may compromise the statistical power and reliability of the study results. More studies needed for update our meta-analysis in the further.

## Conclusion

VEN-based combination therapy demonstrates significant efficacy and a favorable safety profile in patients with AML, potentially providing a more appropriate treatment option. Nevertheless, due to the limited available literature and the presence of heterogeneity and potential publication bias, it is imperative to undertake further prospective studies in the future. These studies are essential for providing more accurate and convincing evidence to guide therapeutic decisions in patients.

## Data availability statement

The original contributions presented in the study are included in the article/**Supplementary Material**. Further inquiries can be directed to the corresponding authors.

## Author contributions

JZ: Writing – original draft. JF: Writing – original draft. TX: Writing – original draft. HZ: Writing – review & editing. RL: Writing – review & editing. YZ: Writing – review & editing. YL: Writing – review & editing. XX: Writing – review & editing. DW: Writing – review & editing. ZJ: Writing – review & editing. FH: Writing – review & editing. RG: Writing – review & editing.

## References

[1] Walker CJ Kohlschmidt J Eisfeld A-K Mrózek K Liyanarachchi S Song C . Genetic characterization and prognostic relevance of acquired uniparental disomies in cytogenetically normal acute myeloid leukemia. Clin Cancer Res. (2019) 25:6524–31. doi: 10.1158/1078-0432.CCR-19-0725 PMC6825549 31375516

[2] Neuendorff NR Loh KP Mims AS Christofyllakis K Soo W-K Bölükbasi B . Anthracycline-related cardiotoxicity in older patients with acute myeloid leukemia: A young siog review paper. Blood Adv. (2020) 4:762–75. doi: 10.1182/bloodadvances.2019000955 PMC7042993 32097461

[3] DiNardo CD Erba HP Freeman SD Wei AH . Acute myeloid leukaemia. Lancet. (2023) 401:2073–86. doi: 10.1016/S0140-6736(23)00108-3 37068505

[4] Wei AH Montesinos P Ivanov V DiNardo CD Novak J Laribi K . Venetoclax plus ldac for newly diagnosed aml ineligible for intensive chemotherapy: A phase 3 randomized placebo-controlled trial. Blood. (2020) 135:2137–45. doi: 10.1182/blood.2020004856 PMC7290090 32219442

[5] Maiti A DiNardo CD Qiao W Kadia TM Jabbour EJ Rausch CR . Ten-day decitabine with venetoclax versus intensive chemotherapy in relapsed or refractory acute myeloid leukemia: A propensity score-matched analysis. Cancer. (2021) 127:4213–20. doi: 10.1002/cncr.33814 PMC8556232 34343352

[6] Griffioen MS de Leeuw DC Janssen JJWM Smit L . Targeting acute myeloid leukemia with venetoclax; biomarkers for sensitivity and rationale for venetoclax-based combination therapies. Cancers (Basel). (2022) 14(14):3456. doi: 10.3390/cancers14143456 35884517 PMC9318140

[7] Wang H Mao L Yang M Qian P Lu H Tong H . Venetoclax plus 3 + 7 daunorubicin and cytarabine chemotherapy as first-line treatment for adults with acute myeloid leukaemia: A multicentre, single-arm, phase 2 trial. Lancet Haematol. (2022) 9:e415–e24. doi: 10.1016/S2352-3026(22)00106-5 35512726

[8] Cherry EM Abbott D Amaya M McMahon C Schwartz M Rosser J . Venetoclax and azacitidine compared with induction chemotherapy for newly diagnosed patients with acute myeloid leukemia. Blood Adv. (2021) 5:5565–73. doi: 10.1182/bloodadvances.2021005538 PMC8714726 34610123

[9] Wei AH Panayiotidis P Montesinos P Laribi K Ivanov V Kim I . 6-month follow-up of viale-C demonstrates improved and durable efficacy in patients with untreated aml ineligible for intensive chemotherapy (141/150). Blood Cancer J. (2021) 11:163. doi: 10.1038/s41408-021-00555-8 34599139 PMC8486817

[10] DiNardo CD Jonas BA Pullarkat V Thirman MJ Garcia JS Wei AH . Azacitidine and venetoclax in previously untreated acute myeloid leukemia. N Engl J Med. (2020) 383:617–29. doi: 10.1056/NEJMoa2012971 32786187

[11] Higgins JPT Altman DG Gøtzsche PC Jüni P Moher D Oxman AD . The cochrane collaboration’s tool for assessing risk of bias in randomised trials. BMJ. (2011) 343:d5928. doi: 10.1136/bmj.d5928 22008217 PMC3196245

[12] Gershon A Ma E Xu T Montez M Naqvi K Ku G . Early real-world first-line treatment with venetoclax plus hmas versus hma monotherapy among patients with aml in a predominately us community setting. Clin Lymphoma Myeloma Leuk. (2023) 23:e222–e31. doi: 10.1016/j.clml.2023.02.002 36925388

[13] Kwag D Cho B-S Bang S-Y Lee JH Min G-J Park S-S . Venetoclax with decitabine versus decitabine monotherapy in elderly acute myeloid leukemia: A propensity score-matched analysis. Blood Cancer J. (2022) 12:169. doi: 10.1038/s41408-022-00770-x 36529771 PMC9760636

[14] Lachowiez CA Reville PK Kantarjian H Jabbour E Borthakur G Daver N . Venetoclax combined with induction chemotherapy in patients with newly diagnosed acute myeloid leukaemia: A post-hoc, propensity score-matched, cohort study. Lancet Haematol. (2022) 9:e350–e60. doi: 10.1016/S2352-3026(22)00076-X PMC9946440 35483396

[15] Maiti A Qiao W Sasaki K Ravandi F Kadia TM Jabbour EJ . Venetoclax with decitabine vs intensive chemotherapy in acute myeloid leukemia: A propensity score matched analysis stratified by risk of treatment-related mortality. Am J Hematol. (2021) 96:282–91. doi: 10.1002/ajh.26061 PMC8128145 33264443

[16] Park S Kwag D Kim TY Lee JH Lee JY Min GJ . A retrospective comparison of salvage intensive chemotherapy versus venetoclax-combined regimen in patients with relapsed/refractory acute myeloid leukemia (Aml). Ther Adv Hematol. (2022) 13:20406207221081637. doi: 10.1177/20406207221081637 35340720 PMC8949776

[17] Shimony S Rozental A Bewersdorf JP Goldberg AD Stein EM Grimshaw AA . Investigational venetoclax combination therapy in acute myeloid leukemia - a systematic review and meta-analysis. Haematologica. (2022) 107:2955–60. doi: 10.3324/haematol.2022.281453 PMC9713559 36453519

[18] DiNardo CD Pratz KW Letai A Jonas BA Wei AH Thirman M . Safety and preliminary efficacy of venetoclax with decitabine or azacitidine in elderly patients with previously untreated acute myeloid leukaemia: A non-randomised, open-label, phase 1b study. Lancet Oncol. (2018) 19:216–28. doi: 10.1016/S1470-2045(18)30010-X 29339097

[19] Lee JB Khan DH Hurren R Xu M Na Y Kang H . Venetoclax enhances T cell-mediated antileukemic activity by increasing ros production. Blood. (2021) 138:234–45. doi: 10.1182/blood.2020009081 PMC8310428 34292323

[20] Thol F Ganser A . Treatment of relapsed acute myeloid leukemia. Curr Treat Options Oncol. (2020) 21:66. doi: 10.1007/s11864-020-00765-5 32601974 PMC7324428

[21] Pei S Pollyea DA Gustafson A Stevens BM Minhajuddin M Fu R . Monocytic subclones confer resistance to venetoclax-based therapy in patients with acute myeloid leukemia. Cancer Discovery. (2020) 10:536–51. doi: 10.1158/2159-8290.CD-19-0710 PMC7124979 31974170

[22] Tsai CH Hou HA Tang JL Liu CY Lin CC Chou WC . Genetic alterations and their clinical implications in older patients with acute myeloid leukemia. Leukemia. (2016) 30:1485–92. doi: 10.1038/leu.2016.65 27055875

[23] Stahl M Menghrajani K Derkach A Chan A Xiao W Glass J . Clinical and molecular predictors of response and survival following venetoclax therapy in relapsed/refractory aml. Blood Adv. (2021) 5:1552–64. doi: 10.1182/bloodadvances.2020003734 PMC7948282 33687434

[24] DiNardo CD Rausch CR Benton C Kadia T Jain N Pemmaraju N . Clinical experience with the bcl2-inhibitor venetoclax in combination therapy for relapsed and refractory acute myeloid leukemia and related myeloid Malignancies. Am J Hematol. (2018) 93:401–7. doi: 10.1002/ajh.25000 29218851

[25] Konopleva M Pollyea DA Potluri J Chyla B Hogdal L Busman T . Efficacy and biological correlates of response in a phase ii study of venetoclax monotherapy in patients with acute myelogenous leukemia. Cancer Discovery. (2016) 6:1106–17. doi: 10.1158/2159-8290.CD-16-0313 PMC5436271 27520294

[26] Huemer F Melchardt T Jansko B Wahida A Jilg S Jost PJ . Durable remissions with venetoclax monotherapy in secondary aml refractory to hypomethylating agents and high expression of bcl-2 and/or bim. Eur J Haematol. (2019) 102:437–41. doi: 10.1111/ejh.13218 PMC6849823 30725494

[27] Ram R Amit O Zuckerman T Gurion R Raanani P Bar-On Y . Venetoclax in patients with acute myeloid leukemia refractory to hypomethylating agents-a multicenter historical prospective study. Ann Hematol. (2019) 98:1927–32. doi: 10.1007/s00277-019-03719-6 31187237

[28] Aldoss I Yang D Pillai R Sanchez JF Mei M Aribi A . Association of leukemia genetics with response to venetoclax and hypomethylating agents in relapsed/refractory acute myeloid leukemia. Am J Hematol. (2019) 94:E253–E5. doi: 10.1002/ajh.25567 PMC8855495 31259427

[29] Gangat N Karrar O Iftikhar M McCullough K Johnson IM Abdelmagid M . Venetoclax and hypomethylating agent combination therapy in newly diagnosed acute myeloid leukemia: genotype signatures for response and survival among 301 consecutive patients. Am J Hematol. (2024) 99:193–202. doi: 10.1002/ajh.27138 38071734

[30] Alkhateeb HB Nanaa A Viswanatha D Foran JM Badar T Sproat L . Genetic features and clinical outcomes of patients with isolated and comutated ddx41-mutated myeloid neoplasms. Blood Adv. (2022) 6:528–32. doi: 10.1182/bloodadvances.2021005738 PMC8791578 34644397

[31] Nanaa A He R Foran JM Badar T Gangat N Pardanani A . Venetoclax plus hypomethylating agents in ddx41-mutated acute myeloid leukaemia and myelodysplastic syndrome: mayo clinic series on 12 patients. Br J Haematol. (2024) 204:171–6. doi: 10.1111/bjh.19105 37710381

[32] Chlon TM Stepanchick E Hershberger CE Daniels NJ Hueneman KM Kuenzi Davis A . Germline ddx41 mutations cause ineffective hematopoiesis and myelodysplasia. Cell Stem Cell. (2021) 28(11):1966–81. doi: 10.1016/j.stem.2021.08.004 PMC8571055 34473945

[33] Senapati J Urrutia S Loghavi S Short NJ Issa GC Maiti A . Venetoclax abrogates the prognostic impact of splicing factor gene mutations in newly diagnosed acute myeloid leukemia. Blood. (2023) 142:1647–57. doi: 10.1182/blood.2023020649 37441846

[34] Lachowiez CA Loghavi S Furudate K Montalban-Bravo G Maiti A Kadia T . Impact of splicing mutations in acute myeloid leukemia treated with hypomethylating agents combined with venetoclax. Blood Adv. (2021) 5:2173–83. doi: 10.1182/bloodadvances.2020004173 PMC8095152 33885753

[35] Crews LA Balaian L Delos Santos NP Leu HS Court AC Lazzari E . Rna splicing modulation selectively impairs leukemia stem cell maintenance in secondary human aml. Cell Stem Cell. (2016) 19:599–612. doi: 10.1016/j.stem.2016.08.003 27570067 PMC5097015

[36] Du Y Li C Yan J . The efficacy and safety of venetoclax and azacytidine combination treatment in patients with acute myeloid leukemia and myelodysplastic syndrome: systematic review and meta-analysis. Hematology. (2023) 28:2198098. doi: 10.1080/16078454.2023.2198098 37036307

