# Supplementary figures and images for: Venetoclax combined chemotherapy versus chemotherapy alone for acute myeloid leukemia: a systematic review and meta-analysis
Source: Front Oncol. 2024 Mar 26;14:1361988. doi: 10.3389/fonc.2024.1361988 (PMC11002170; doi:10.3389/fonc.2024.1361988)

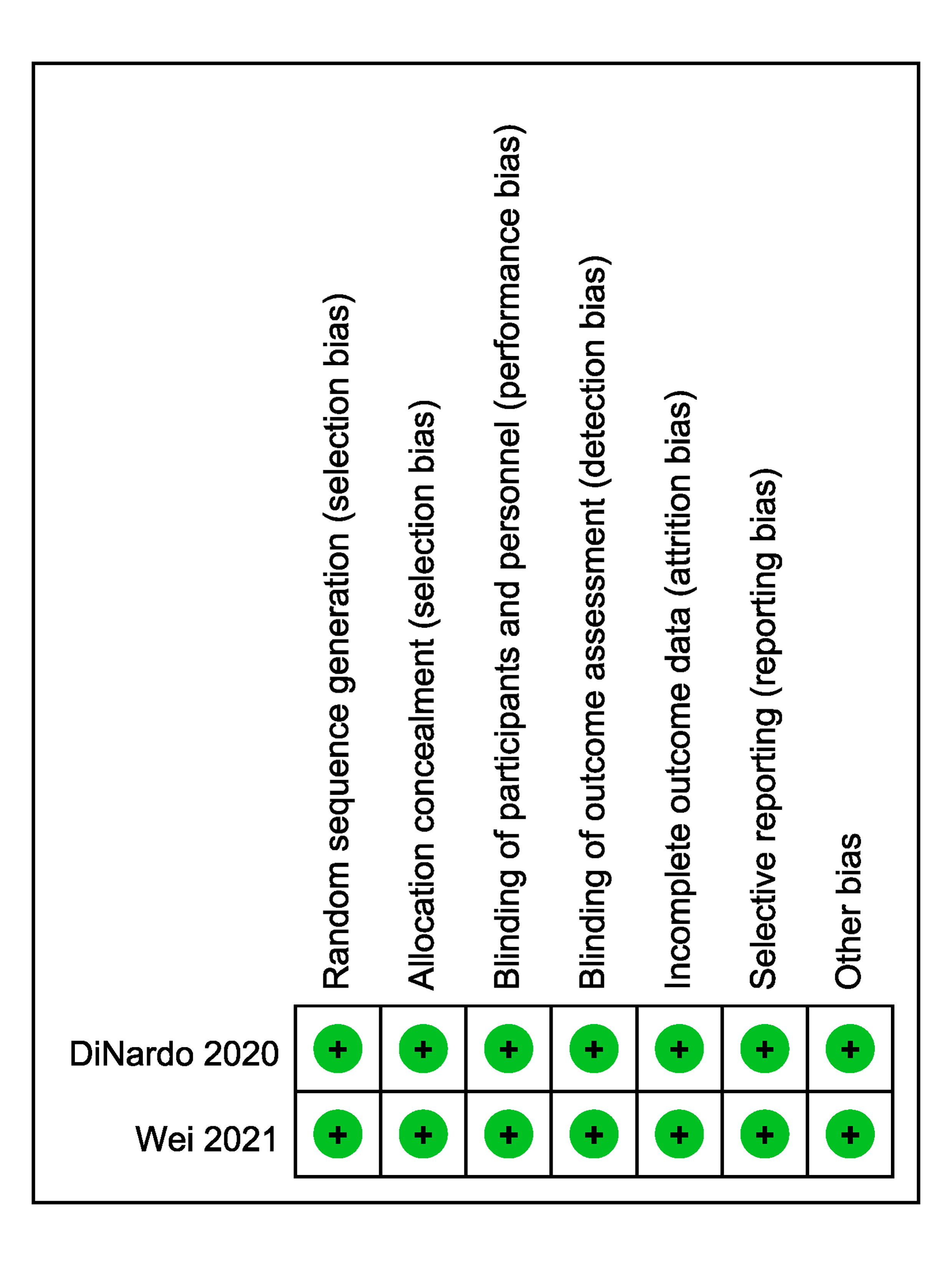

Supplement: Supplementary file 1 [file Image_1.tiff]
