# Supplementary material for: Venetoclax combined chemotherapy versus chemotherapy alone for acute myeloid leukemia: a systematic review and meta-analysis
Source: Front Oncol. 2024 Mar 26;14:1361988. doi: 10.3389/fonc.2024.1361988 (PMC11002170; doi:10.3389/fonc.2024.1361988)
Supplement: Supplementary file 2 [file Table_1.docx]

Supplementary Table S1. The cytogenetic/molecular/ELN risk information of patients

| Study | ELN risks (Venetoclax+chemo/Chemo, n) | | | Cytogenetics (Venetoclax+chemo/Chemo, n) | | | Mutation status (Venetoclax+chemo/Chemo, n) | | | | | |
| --- | --- | --- | --- | --- | --- | --- | --- | --- | --- | --- | --- | --- |
|  | Favorable | Intermediate | Adverse | Favorable | Intermediate | Adverse | FLT3-ITD | IDH1/2 | NPM1 | TP53 | ASXL1 | RUNX1 |
| Cherry 2021 | 93/60 | 24/23 | 24/24 | NA | NA | NA | 24/40 | 39/25 | 33/42 | 25/4 | 36/11 | 23/10 |
| DiNardo 2020 | NA | NA | NA | 0/0 | 182/89 | 104/56 | NA | 61/28 | 27/17 | 38/14 | NA | NA |
| Gershon 2023 | 68/33 | 153/84 | 251/67 | NA | NA | NA | NA | NA | NA | NA | NA | NA |
| Kwag 2022 | NA | NA | NA | 14/12 | 50/51 | 17/17 | 6/9 | NA | 12/17 |  | NA | NA |
| Lachowiez 2022 | 21/32 | 29/67 | 35/95 | 0/1 | 58/128 | 22/58 | 12/58 | 17/35 | 20/51 | 5/15 | 8/20 | 11/9 |
| Maiti 2021a | 10/11 | 12/30 | 43/89 | 0/0 | 41/80 | 24/50 | NA | 11/28 | 14/15 | 18/32 | 10/14 | 12/22 |
| Maiti 2021b | 19/14 | 11/17 | 55/54 | 0/0 | 44/33 | 40/52 | NA |  |  |  |  |  |
| Park 2022 | 10/19 | 23/26 | 21/44 | 2/5 | 39/59 | 13/25 | 14/24 | 13/11 | 10/16 | 5/4 | 1/7 | 7/17 |
| Wei 2021 | NA | NA | NA | 1/3 | 90/43 | 47/20 | NA |  |  |  |  |  |
