# Supplementary material for: Venetoclax combined chemotherapy versus chemotherapy alone for acute myeloid leukemia: a systematic review and meta-analysis
Source: Front Oncol. 2024 Mar 26;14:1361988. doi: 10.3389/fonc.2024.1361988 (PMC11002170; doi:10.3389/fonc.2024.1361988)
Supplement: Supplementary file 3 [file Table_2.docx]

| Supplementary Table S2. Quality evaluation of the eligible studies with Newcastle–Ottawa scale. | | | | | | | | | |
| --- | --- | --- | --- | --- | --- | --- | --- | --- | --- |
| Study | Selection | | | | Comparability | | Outcome | | |
|  | Representative-ness | Selection of  non-exposed | Ascertainment  of exposure | Outcome not present at start | Comparability on most important factors | Comparability on other risk factors | Assessment of outcome | Long enough follow-up (median≥1 year) | Adequacy  (completeness) of follow-up |
| Cherry 2021 | * | * | * | * | - | - | * | * | * |
| Gershon 2023 | * | * | * | * | - | - | * | - | * |
| Kwag 2022 | * | * | * | * | * | * | * | - | * |
| Lachowiez 2022 | * | * | * | * | * | - | * | * | * |
| Maiti 2021a | * | * | * | * | * | * | * | * | * |
| Maiti 2021b | * | * | * | * | * | - | * | * | * |
| Park 2022 | * | * | * | * | * | - | * | * | * |
| *indicates criterion met; - indicates significant of criterion not met. | | | | | | | | | |
