# Supplementary material for: Venetoclax combined chemotherapy versus chemotherapy alone for acute myeloid leukemia: a systematic review and meta-analysis
Source: Front Oncol. 2024 Mar 26;14:1361988. doi: 10.3389/fonc.2024.1361988 (PMC11002170; doi:10.3389/fonc.2024.1361988)
Supplement: Supplementary file 4 [file DataSheet_1.docx]

Data Sheet 1. The comprehensive search strategy of “Venetoclax combined chemotherapy versus chemotherapy alone for acute myeloid leukemia: a systematic review and meta-analysis”

**Venetoclax**

GDC-0199

RG-7601

RG7601

ABT-199

Venclexta

Chemotherapy

**Leukemia, Myeloid, Acute**

Acute Myeloid Leukemia

Acute Myeloid Leukemias

Leukemias, Acute Myeloid

Myeloid Leukemias, Acute

ANLL

Leukemia, Acute Myelogenous

Leukemia, Acute Myeloid

Leukemia, Myeloblastic, Acute

Leukemia, Myelocytic, Acute

Leukemia, Myelogenous, Acute

Leukemia, Nonlymphoblastic, Acute

Leukemia, Nonlymphocytic, Acute

Myeloblastic Leukemia, Acute

Acute Myeloblastic Leukemia

Acute Myeloblastic Leukemias

Leukemia, Acute Myeloblastic

Leukemias, Acute Myeloblastic

Myeloblastic Leukemias, Acute

Myelocytic Leukemia, Acute

Acute Myelocytic Leukemia

Acute Myelocytic Leukemias

Leukemia, Acute Myelocytic

Leukemias, Acute Myelocytic

Myelocytic Leukemias, Acute

Myelogenous Leukemia, Acute

Myeloid Leukemia, Acute

Nonlymphoblastic Leukemia, Acute

Acute Nonlymphoblastic Leukemia

Acute Nonlymphoblastic Leukemias

Leukemia, Acute Nonlymphoblastic

Leukemias, Acute Nonlymphoblastic

Nonlymphoblastic Leukemias, Acute

Nonlymphocytic Leukemia, Acute

Acute Nonlymphocytic Leukemia

Acute Nonlymphocytic Leukemias

Leukemia, Acute Nonlymphocytic

Leukemias, Acute Nonlymphocytic

Nonlymphocytic Leukemias, Acute

Acute Myelogenous Leukemia

Acute Myelogenous Leukemias

Leukemias, Acute Myelogenous

Myelogenous Leukemias, Acute

Myeloid Leukemia, Acute, M1

Leukemia, Myeloid, Acute, M1

Acute Myeloid Leukemia without Maturation

Leukemia, Myeloid, Acute, M2

Myeloid Leukemia, Acute, M2

Acute Myeloid Leukemia with Maturation

Pubmed-794

((("venetoclax" [Supplementary Concept]) OR ((((((Venetoclax) OR (GDC-0199)) OR (RG-7601)) OR (RG7601)) OR (ABT-199)) OR (Venclexta))) AND (("Leukemia, Myeloid, Acute"[Mesh]) OR ((((((((((((((((((((((((((((((((((((((((((((((((Acute Myeloid Leukemia) OR (Acute Myeloid Leukemias)) OR (Leukemias, Acute Myeloid)) OR (Myeloid Leukemias, Acute)) OR (ANLL)) OR (Leukemia, Acute Myelogenous)) OR (Leukemia, Acute Myeloid)) OR (Leukemia, Myeloblastic, Acute)) OR (Leukemia, Myelocytic, Acute)) OR (Leukemia, Myelogenous, Acute)) OR (Leukemia, Nonlymphoblastic, Acute)) OR (Leukemia, Nonlymphocytic, Acute)) OR (Myeloblastic Leukemia, Acute)) OR (Acute Myeloblastic Leukemia)) OR (Acute Myeloblastic Leukemias)) OR (Leukemia, Acute Myeloblastic)) OR (Leukemias, Acute Myeloblastic)) OR (Myeloblastic Leukemias, Acute)) OR (Myelocytic Leukemia, Acute)) OR (Acute Myelocytic Leukemia)) OR (Acute Myelocytic Leukemias)) OR (Leukemia, Acute Myelocytic)) OR (Leukemias, Acute Myelocytic)) OR (Myelocytic Leukemias, Acute)) OR (Myelogenous Leukemia, Acute)) OR (Myeloid Leukemia, Acute)) OR (Nonlymphoblastic Leukemia, Acute)) OR (Acute Nonlymphoblastic Leukemia)) OR (Acute Nonlymphoblastic Leukemias)) OR (Leukemia, Acute Nonlymphoblastic)) OR (Leukemias, Acute Nonlymphoblastic)) OR (Nonlymphoblastic Leukemias, Acute)) OR (Nonlymphocytic Leukemia, Acute)) OR (Acute Nonlymphocytic Leukemia)) OR (Acute Nonlymphocytic Leukemias)) OR (Leukemia, Acute Nonlymphocytic)) OR (Leukemias, Acute Nonlymphocytic)) OR (Nonlymphocytic Leukemias, Acute)) OR (Acute Myelogenous Leukemia)) OR (Acute Myelogenous Leukemias)) OR (Leukemias, Acute Myelogenous)) OR (Myelogenous Leukemias, Acute)) OR (Myeloid Leukemia, Acute, M1)) OR (Leukemia, Myeloid, Acute, M1)) OR (Acute Myeloid Leukemia without Maturation)) OR (Leukemia, Myeloid, Acute, M2)) OR (Myeloid Leukemia, Acute, M2)) OR (Acute Myeloid Leukemia with Maturation)))) AND (Chemotherapy)

Embase-1081


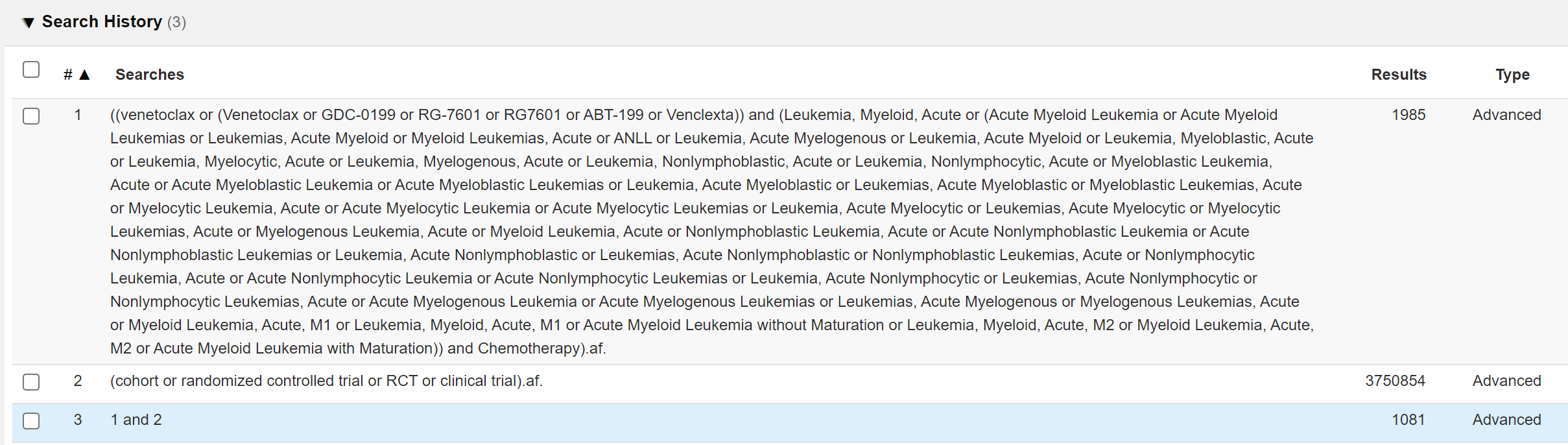


Cochrane-120


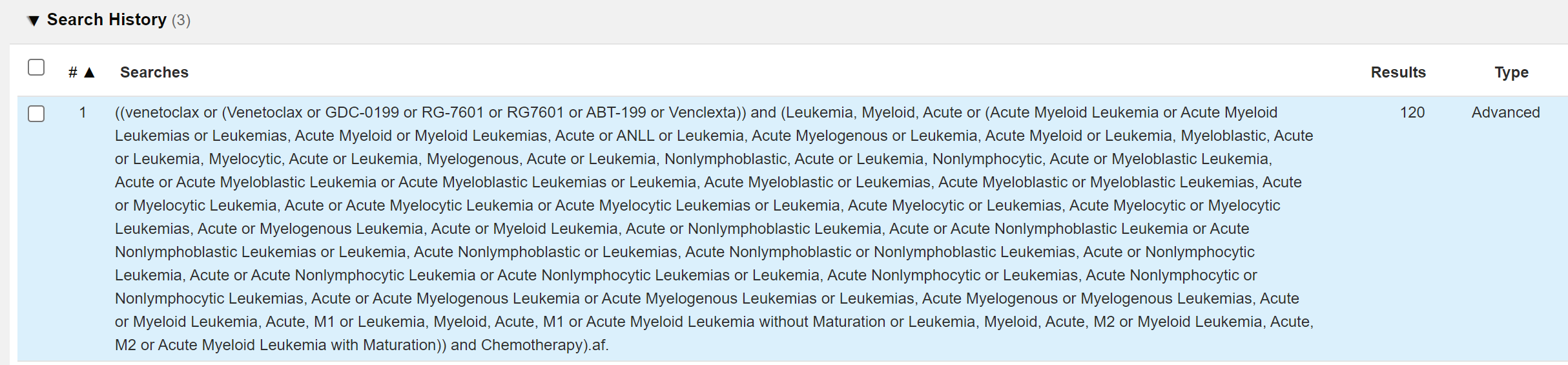


WOS-611

| # | Search Query | Database | Results |
| --- | --- | --- | --- |
| 1 | (((venetoclax) OR ((((((Venetoclax) OR (GDC-0199)) OR (RG-7601)) OR (RG7601)) OR (ABT-199)) OR (Venclexta))) AND ((Leukemia, Myeloid, Acute) OR ((((((((((((((((((((((((((((((((((((((((((((((((Acute Myeloid Leukemia) OR (Acute Myeloid Leukemias)) OR (Leukemias, Acute Myeloid)) OR (Myeloid Leukemias, Acute)) OR (ANLL)) OR (Leukemia, Acute Myelogenous)) OR (Leukemia, Acute Myeloid)) OR (Leukemia, Myeloblastic, Acute)) OR (Leukemia, Myelocytic, Acute)) OR (Leukemia, Myelogenous, Acute)) OR (Leukemia, Nonlymphoblastic, Acute)) OR (Leukemia, Nonlymphocytic, Acute)) OR (Myeloblastic Leukemia, Acute)) OR (Acute Myeloblastic Leukemia)) OR (Acute Myeloblastic Leukemias)) OR (Leukemia, Acute Myeloblastic)) OR (Leukemias, Acute Myeloblastic)) OR (Myeloblastic Leukemias, Acute)) OR (Myelocytic Leukemia, Acute)) OR (Acute Myelocytic Leukemia)) OR (Acute Myelocytic Leukemias)) OR (Leukemia, Acute Myelocytic)) OR (Leukemias, Acute Myelocytic)) OR (Myelocytic Leukemias, Acute)) OR (Myelogenous Leukemia, Acute)) OR (Myeloid Leukemia, Acute)) OR (Nonlymphoblastic Leukemia, Acute)) OR (Acute Nonlymphoblastic Leukemia)) OR (Acute Nonlymphoblastic Leukemias)) OR (Leukemia, Acute Nonlymphoblastic)) OR (Leukemias, Acute Nonlymphoblastic)) OR (Nonlymphoblastic Leukemias, Acute)) OR (Nonlymphocytic Leukemia, Acute)) OR (Acute Nonlymphocytic Leukemia)) OR (Acute Nonlymphocytic Leukemias)) OR (Leukemia, Acute Nonlymphocytic)) OR (Leukemias, Acute Nonlymphocytic)) OR (Nonlymphocytic Leukemias, Acute)) OR (Acute Myelogenous Leukemia)) OR (Acute Myelogenous Leukemias)) OR (Leukemias, Acute Myelogenous)) OR (Myelogenous Leukemias, Acute)) OR (Myeloid Leukemia, Acute, M1)) OR (Leukemia, Myeloid, Acute, M1)) OR (Acute Myeloid Leukemia without Maturation)) OR (Leukemia, Myeloid, Acute, M2)) OR (Myeloid Leukemia, Acute, M2)) OR (Acute Myeloid Leukemia with Maturation)))) AND (Chemotherapy) (Topic) and Preprint Citation Index (Exclude – Database) | All Databases | 1231 |
| 2 | cohort (Topic) OR RCT (Topic) OR randomized controlled trial (Topic) OR clinical trial (Topic) and Preprint Citation Index (Exclude – Database) | All Databases | 2915546 |
| 3 | #2 AND #1 and Preprint Citation Index (Exclude – Database) | All Databases | 611 |
